# Food Insecurity and Mental Health among Females in High-Income Countries

**DOI:** 10.3390/ijerph15071424

**Published:** 2018-07-06

**Authors:** Merryn Maynard, Lesley Andrade, Sara Packull-McCormick, Christopher M. Perlman, Cesar Leos-Toro, Sharon I. Kirkpatrick

**Affiliations:** 1 Meal Exchange Canada, Toronto, ON M5V 3A8, Canada; 2 School of Public Health and Health Systems, University of Waterloo, Waterloo, ON N2L 3G1, Canada; landrade@uwaterloo.ca (L.A.); srpackul@uwaterloo.ca (S.P.M.); chris.perlman@uwaterloo.ca (C.M.P.); cesar.leos-toro@uwaterloo.ca (C.L.-T.)

**Keywords:** food insecurity, mental health, depression, women, scoping review

## Abstract

Food insecurity is a persistent concern in high-income countries, and has been associated with poor mental health, particularly among females. We conducted a scoping review to characterize the state of the evidence on food insecurity and mental health among women in high-income countries. The research databases PubMed, EMBASE, and psycINFO were searched using keywords capturing food insecurity, mental health, and women. Thirty-nine articles (representing 31 unique studies/surveys) were identified. Three-quarters of the articles drew upon data from a version of the United States Department of Agriculture Household Food Security Survey Module. A range of mental health measures were used, most commonly to measure depression and depressive symptoms, but also anxiety and stress. Most research was cross-sectional and showed associations between depression and food insecurity; longitudinal analyses suggested bidirectional relationships (with food insecurity increasing the risk of depressive symptoms or diagnosis, or depression predicting food insecurity). Several articles focused on vulnerable subgroups, such as pregnant women and mothers, women at risk of homelessness, refugees, and those who had been exposed to violence or substance abuse. Overall, this review supports a link between food insecurity and mental health (and other factors, such as housing circumstances and exposure to violence) among women in high-income countries and underscores the need for comprehensive policies and programs that recognize complex links among public health challenges.

## 1. Introduction

Food insecurity is a growing and persistent concern in high-income countries [1,2]. In North America, rates of household food insecurity have remained stable or risen in the last several years [3,4]. High rates have also been documented in the UK and Australia [5,6]. According to the Food and Agriculture Organization, “food security exists when all people, at all times, have physical, social, and economic access to sufficient safe and nutritious food that meets their dietary needs and food preferences for an active and healthy life” [7].Conceptualizations of food insecurity in high-income countries primarily focus on the economic aspect; for example, the Household Food Security Survey Module (HFSSM) [8], which is commonly used in the United States and Canada, measures uncertain or inadequate access to food due to financial constraints. This conceptualization aligns with literature linking vulnerability to food insecurity to high rates of poverty, particularly among population subgroups, such as single-parent households, racial/ethnic minorities, and those relying on social assistance [2,3,4,9,10,11,12,13]. 

Among population subgroups in high-income countries, food insecurity has been shown to be associated with compromised nutrition [14], poor general health, and a myriad of chronic health conditions [15,16]. Food insecurity has also been shown to be a marker of poor mental health, with studies identifying associations with mood and anxiety disorders and suicidal ideation, particularly among women [16,17,18]. Indeed, severity of household food insecurity appears to be linked with poor mental health in a dose–response manner, with experiences of severe food insecurity representing extreme chronic stress [19] and possibly acting as an independent determinant of suicidal ideation [20]. 

The relationship between food insecurity and poor mental health among women is of particular concern given that they are disproportionately impacted by food insecurity [2,3,4,21]. Women are overrepresented among low-income groups compared to men, with visible minority women and single mothers experiencing high rates of poverty in Canada and the United States [9,10,11]. Further, the existing literature suggests that women may be particularly vulnerable to poor mental health in conjunction with poverty and food insecurity [12] and for women with children, that the stress associated with these experiences has possible ripple effects, negatively impacting their children’s physical and mental health as well [13].

To identify future research needs and inform policy and program responses, we conducted a scoping review to examine the state of the literature on food insecurity and mental health among women living in high-income countries. 

## 2. Materials and Methods 

The scoping review was conducted according to steps outlined by Arksey and O’Malley [22]. Scoping reviews, which use systematic search techniques, are appropriate when the aim is to address a broad question, such as querying the state of the evidence on a topic (especially when study designs may vary) and identifying gaps in that evidence [22] to inform future research and practice. As per Arksey and O’Malley [22], steps in the process include identifying the research question, identifying relevant studies, study selection, charting the data, and collating, summarizing, and reporting the results. Reporting follows the PRISMA guidelines [23].

### 2.1. Identifying Relevant Studies

**Figure 1:**
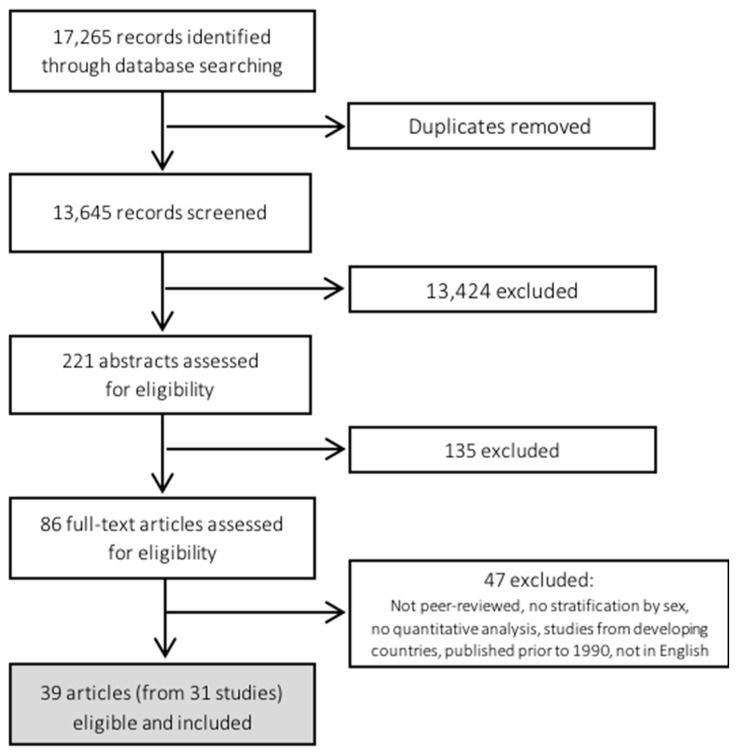
Overview of identification and screening of records for scoping review of literature on food insecurity and mental health among women in high-income countries.

The systematic search, developed in consultation with a librarian who is an expert in systematic searching, was conducted using the research databases PubMed, EMBASE, and psycINFO to capture records published up to May 2016. Given the range of possible mental health conditions, the search strategy was quite broad. Key words and Medical Subject Headings (MeSH) included “food” OR “nutrition” OR “diet” AND “security” OR “insecurity” OR “insufficiency” OR “scarcity” OR “*adequacy” OR “hunger” OR “poverty” OR “food supply” OR “nutritional requirements/status” AND “anxiety” OR “depression” OR “mental health” OR “mental health disorder” OR “mental health illness” OR “psychosis” OR “emotional disorder” OR “mania” OR “mental disease” OR “phobia” OR “mental disturbance/health/psychology”. The key words and MESH headings to capture women included “women” OR “woman” OR “female” OR “pregnancy” OR “sex factors” OR “women’s rights” OR “mothers” OR “girl” (note: * indicates a wildcard, which allows searching a range of terms related to a root word). The initial search elicited a total of 13,645 citations (excluding duplicates) (Figure 1). 

### 2.2. Study Selection

Articles deemed eligible quantitatively examined associations between food insecurity and indicators of mental health, with a focus on females in high-income countries; studies that included both males and females but reported analyses stratified by sex were also considered. Specific criteria related to age were not applied, allowing consideration of studies reporting on adolescent girls as well as women. Studies published since 1990 (to provide insights into relatively recent research on the topic of food insecurity) were considered. 

An initial screening of titles and abstracts was conducted by one author (S.P.-M.) to identify potentially relevant peer-reviewed articles that addressed food insecurity and health, leaving 221 citations for further review (Figure 1). Abstracts for these 221 citations were screened independently by a second author (M.M. or S.I.K.) and discrepancies resolved, leaving 86 citations for full-text review. After full-text screening (conducted independently by two authors), 39 articles remained, representing 31 unique studies/surveys. Separate articles making use of data from the same study or survey were examined and charted to identify salient characteristics related to measurement of food security and mental health and the examination of associations between the two. 

### 2.3. Charting the Data

A data abstraction form guided extraction of the characteristics of interest, including study setting and population, study design, main study objectives, measures used to assess food security and mental health and specific mental health states considered, and analytic approach and findings. 

### 2.4. Collating, Summarizing, and Reporting the Results

The abstracted data were assessed in terms of patterns in measures and tools used and associations between food insecurity and depression (the most frequently examined mental health measure) and other mental health markers. Given that we conducted a scoping rather than a systematic review, formal quality appraisal of studies was not conducted [22]. However, in addition to synthesizing the evidence emerging from this literature, we comment on the characteristics of the available research, in terms of study design for example, to inform future research.

## 3. Results

### 3.1. Overview of Included Articles

The characteristics of the 39 articles are outlined in Appendix A. Over half (*n =* 23) were published from 2010 on [15,16,17,24,25,26,27,28,29,30,31,32,33,34,35,36,37,38,39,40,41,42,43]. The majority (*n =* 34) analyzed data from studies conducted in the United States, three focused on studies conducted in Canada [16,17,32], one was focused on a sample in New Zealand [43], and one was conducted in England [44]. Twenty-eight articles reported on cross-sectional analyses (one also included qualitative data collection [28]) and eleven reported longitudinal analyses (one included qualitative data collection [45]) (Appendix A). Although all studies assessed the association between food insecurity and a mental health condition or state in some manner, the particular research questions and analytic approaches varied. Some studies examined food insecurity and mental health among general samples of the population, whereas others focused on particularly vulnerable population subgroups or sought to assess the feasibility or other properties of tools. Half (*n* = 20) focused on mothers or caregivers, another five studied pregnant women, and several focused on other specific subpopulations, including rural women, those living with disabilities, older women, refugees, women experiencing insecure housing or homelessness, and women at risk for HIV (Appendix A). 

### 3.2. Food Insecurity Measures

Three-quarters (*n* = 30) of the reviewed articles drew upon data collected using a version of the Household Food Security Survey Module (HFSSM), developed by the United States Department of Agriculture [8] (Table 1). The full HFSSM contains 18 items and yields a single score indicating the severity of household food insecurity over the past 12 months or 30 days; ten items refer to adults and eight refer to children in the household [8]. Scores are typically used to categorize households as food secure or food insecure with different levels of severity (since a review of the measure conducted in the early 2000s [46], the categories of food insecurity have been referred to as low and very low food security, replacing earlier labels of food insecure with/without hunger). The HFSSM was compared to household food expenditures and income [8] and associated with compromised dietary intakes [14], supporting its validity in capturing constrained food access due to inadequate finances. Fourteen articles drew upon abbreviated versions of HFSSM, including the six-item subset developed by USDA and the ten adult-referenced items, as well as other adaptations (Table 1). 

One article reported on data using a single item drawn from the 12-item Radimer–Cornell scale [47], and another used data collected using the Community Childhood Hunger Identification Project (CCHIP) instrument [48]. Both the Radimer–Cornell and CCHIP tools are used to categorize food security status and were shown to have good specificity and sensitivity compared to evaluations of food security status based on household food inventories, dietary recall data, and other measures among a sample of women living with children in rural New York [49]. These tools were drawn upon in the development of the HFSSM [8]. 

Three articles drew upon data collected using a single item from the National Health and Nutrition Examination Survey-III (NHANES-III) to assess food insufficiency (defined as “an inadequate amount of food intake due to a lack of money or resources”) [50]. As opposed to more comprehensive instruments, measures of food insufficiency are less detailed and may misclassify some households [49,51]. Finally, four articles drew upon data from other single- or multi-measures adapted from prior literature (Table 1). 

### 3.3. Mental Health Measures

Depression and depressive symptoms were the most prevalent mental health states assessed. Associations between food insecurity and depression were examined in 36 articles (Appendix A). Ten articles drew upon measures assessing clinical diagnoses, while the remainder relied upon self-reported symptoms. 

Measures are described in Table 2, along with information about their validation. In reviewed articles, authors sometimes noted that measures have been tested for psychometric properties such as internal consistency, in some cases, in the context of the particular study (Appendix A). Data from the short form of the World Health Organization World Mental Health Composite International Diagnostic Interview (CIDI) [70] were drawn upon to establish a clinical diagnosis of depression or anxiety in six articles. To assess depressive symptoms, the Centre for Epidemiologic Studies Depression Scale (CES-D) [71] was used most frequently, drawn upon in 14 articles. For anxiety, one article drew upon data from Spielberger’s Trait Anxiety Inventory [72] and another the Hopkins Symptom Checklist Subscale (HSCL) [73]. Some measures targeted specific life stages such as pregnancy and older age; for example, maternal depressive symptoms were assessed with the Kemper three-item screen [74] and the Edinburgh Postpartum Depression Scale [75], while depressive symptoms among older women were assessed using the Geriatric Depression Scale [76]. 

Various other mental health markers were measured, including perceived control over one’s life, perceived stress, quality of life, self-esteem, mastery, general mental health, psychosis, substance abuse, post-traumatic stress disorder, and disordered eating (Appendix A).

### 3.4. Overview of Findings on Food Insecurity and Mental Health

The majority of cross-sectional analyses examining depression and food insecurity (or food insufficiency) reported some form of association deemed to be significant [16,28,29,32,34,35,36,38,39,40,41,42,43,44,54,56,57,58,59,60,63,64,68,69,85]. Several longitudinal analyses likewise observed relationships between depression and food insecurity, with food insecurity increasing the risk of experiencing depressive symptoms or a depression diagnosis [44,53,62], or changes in food insecurity associated with changes in depression [62]. For example, a longitudinal analysis of data from 8693 parent–child dyads by Bronte-Tinkew et al. [53] found that mothers affected by food insecurity were more likely to report depressive symptoms compared to food-secure mothers. Some authors reported that the relationship functioned in the opposite direction, with depression leading to food insecurity [15,24,25,26,45], or was bidirectional [55]. For example, Garg et al., who analyzed data from the Early Childhood Longitudinal Study Birth Cohort (*n* = 2917), found that mothers who experienced depression were at greater risk of remaining food insecure over time compared to mothers without depression [25]. Food insecurity and depression were also investigated in relation to other markers of material deprivation; for example, Corman et al. [24] found that women who experienced a major depressive episode at baseline had greater odds of experiencing food insecurity and inadequate housing at follow-up.

Several articles focused on pregnant women and revealed associations between prenatal and postpartum depression and food insecurity [33,36,42,56,60]. Food-insecure pregnant women were at increased risk of experiencing prenatal depressive symptoms compared to their food-secure counterparts [33,36]. Although a comprehensive measure of food insecurity was not used, Birmingham et al. [42] tested depression screening methods in a cross-sectional analysis of 195 mothers of newborns and found that those who had concerns about food were 5.5 times more likely to have a positive postpartum depression screen result.

Anxiety and stress were associated with food insecurity in multiple studies [16,17,32,56,59]. Analyses of cross-sectional data from the 2007–2008 Canadian Community Health Survey (CCHS) by Tarasuk et al. (*n* = 77,053) [16] and Muldoon et al. (*n* = 5588) [32] indicated that severe food insecurity and a self-reported diagnosis of mood or anxiety disorders were associated among women. Siefert et al. [64] found an association between food insecurity and generalized anxiety disorder in a cross-sectional study of 724 US women receiving welfare, but the relationship was not significant when covariates were taken into account. In two studies, one cross-sectional (*n* = 606) [56] and one longitudinal (*n* = 526) [27], Laraia et al. found that food-insecure pregnant women had higher perceived stress compared to food-secure women, and those who had experienced any level of food insecurity during pregnancy or at three months postpartum were more likely to have high perceived stress scores at 12 months postpartum. Martin et al. [17] investigated perceived stress among Canadian adults and found that the prevalence of high levels of stress increased with lower food security status. However, Trapp et al. [31] explored food insecurity among a group of 222 low-income mothers and their children in a cross-sectional analysis and found that levels of perceived stress did not differ between food-insecure and food-secure groups.

Three recent articles explored disordered or emotional eating among women experiencing food insecurity [27,37,39]. Laraia et al. [27] and Sharpe et al. [39] found bivariate associations between food insecurity and disordered or emotional eating; however, in models adjusted for sociodemographic characteristics, Laraia et al. [27] did not observe significant associations between food insecurity and disordered eating behaviors. Dressler et al. [37] examined associations between emotional eating and depression and suggested that emotional eating may mediate associations among food insecurity, mental health, and other food-related outcomes, such as dietary intakes and weight status.

Moreover, some studies examined multiple mental and physical health conditions suggesting comorbid physical and mental health problems increased vulnerability to food insecurity [16,34] and that food insecurity increased vulnerability to poor physical and mental health [41,69]. There was also a focus on implications for others, including children, in the household. For example, Bronte-Tinkew et al. [53] found that mothers living in food-insecure households reported high rates of depression, which was correlated with fair and poor health in children.

Given that the precise focus of the studies varied, a range of covariates was examined. Several studies examined various forms of social support [15,17,35,52,60,63]. Instrumental social support (e.g., ability to borrow money, help with childcare and transportation) was examined in a study conducted by the Detroit Centre for Oral Health Disparities. Cross-sectional analyses by Siefert et al. [63] (*n* = 824) indicated that the effect of food insufficiency on depression could be reduced with the availability of instrumental social support, while Ajrouch et al. [35] (*n* = 736) found that this protective effect was dampened when respondents experienced high levels of food insecurity-related stress. Using cross-sectional Canadian data, Martin et al. [17] (*n* = 100,401) found associations between food insecurity and feelings of community belonging; for example, the prevalences of living in severely food-insecure households were 18% and 25.6% among women reporting high and low community belonging, respectively. In a cross-sectional analysis, Wehler et al. [52] (*n* = 354) found that financial social support from a sibling reduced the odds of mothers experiencing hunger but did not reduce the odds of children in the same household experiencing hunger. Further, Hanson and Olson [15] (*n* = 225) found that parenting social support (e.g., having someone to talk to and having help in an emergency) did not reduce the odds of a household experiencing persistent vs. discontinuous food insecurity over a period of three years.

The role of childhood and adulthood adverse experiences, including abuse, was also examined. In multivariable models, Wehler et al. [52] found that sexual abuse in childhood increased the odds of adult hunger, and that this appeared to be mediated by experiences of intimate partner violence in adulthood. Sun et al. [30] examined Adverse Childhood Experiences, including abuse, neglect, and household dysfunction, and found that mothers reporting four or more adverse experiences were more likely to report food insecurity, with adjustment for demographic factors. In bivariate analyses, Harrison et al. [60] found that each of food insecurity, intimate partner violence and depressive symptoms were correlated. In multivariable models accounting for demographic factors, Melchior et al. [44] found that intimate partner violence was higher among women who had reported indications of food insecurity two years prior.

## 4. Discussion

Overall, the evidence reviewed here supports a link between food insecurity and compromised mental health among women in high-income countries. Although longitudinal data were limited, associations between food insecurity and depression appear to operate in both directions. There are multiple plausible potential pathways by which food insecurity and poor mental health may be linked. The experience of food insecurity itself is characterized by worry and anxiety about the household food supply. Toxic stress, which refers to chronic and unyielding stress without adequate social and environmental supports [13], may be one pathway through which food insecurity and mental health are intertwined. Depending on the availability and regularity of finances, periods of household food insecurity can occur repeatedly or chronically; households in the United States that were food insecure in 2016 experienced food insecurity in seven months on average [3]. Therefore, food insecurity may represent a chronic stressor that could contribute to the development of poor mental health. Conversely, a mental health condition could inhibit an individual from maintaining steady employment, thereby increasing vulnerability to food insecurity. Further, Seligman and Schillinger [86] posit that the relationship between food insecurity and poor health is cyclical; food insecurity increases the likelihood of trade-offs in food choices among those who receive low income and challenges the self-management of health conditions. Poor self-management results in higher health care and medication costs for the individual, which further contribute to financial instability and food insecurity [86]. Once an individual enters this cycle, it may be very difficult to exit, particularly in countries where there are disparities in access to health care and social supports, impacting access. Additionally, studies found an association between instances of abuse and depression and food insecurity [26,44,52,60]. The early life stress hypothesis argues that stressors experienced during key developmental periods can enhance vulnerability to mental health outcomes in adult life [87].

The majority of the available literature is cross-sectional, and further longitudinal research could shed light on the nature of the observed relationships and factors that underlie them. For example, research is needed to examine the interconnections among various markers of mental health and experiences of food insecurity across the lifespan, as well as to further examine the influence of potential mediating factors, such as social support or experiences of abuse. Many existing studies have focused on women with children, and pregnant women have also been investigated. A population of growing interest in regards to food insecurity is postsecondary students [88,89,90,91]; given that this is a life stage during which vulnerability to poor mental health is also high [89,92,93], research examining the root causes of both issues and how they interact is of public health importance. At the other end of the spectrum, we also identified little research focused on older women.

Food insecurity is a complex and multidimensional phenomenon [51,94] and its measurement is also complex. Many of the reviewed studies relied upon data from the HFSSM, or an adaptation, to assess food security. The HFSSM is considered the standard in household food insecurity measurement in North America and is used widely in research and surveillance [3,4]. While this tool provides an indicator of quantitative deprivation, it focuses on economic access to food and does not capture aspects that are likely to be relevant to mental health, such as the social acceptability of food acquisition strategies [51]. For example, Hamelin et al. have described alienation that accompanies lack of access to adequate food [67], as well as the social implications [95]. Nonetheless, the HFSSM has been widely-used and, within the North American context, provides data that are comparable to those from national surveys [4,8,21]. The Household Food Insecurity Access Scale (HFIAS) [94] is a standardized tool that uses similar questions as the HFSSM and is designed to differentiate food-secure from food-insecure households across cultural contexts; this tool may be appropriate depending on the setting and populations of interest. Whenever feasible, a comprehensive tool is recommended over single or brief measures that may not accurately classify households and cannot provide insights into severity of food insecurity (thus potentially missing the opportunity to shed insights into those who are most vulnerable). Additionally, studies using mixed methods can generate unique information not yielded by a standardized measure such as the HFSSM.

There was greater variety in measures used to assess mental health compared with those used to determine household food security status, the majority involving screening for depressive symptoms, along with diagnostic measures that use more stringent criteria. Many authors noted that these tools had been tested and are widely used, but the range of tools used makes it difficult to compare across studies. As with food insecurity, abbreviated measures, such as those assessing depression and depressive symptoms, may have been limited in sensitivity and specificity compared to full measures, potentially dampening observed relationships or creating spurious effects. While the use of comprehensive measures and greater standardization of tools used to assess depression and other mental health conditions may allow for greater comparability across this body of literature and more robust inferences, it is critical for any study that the measure be well suited to the research question and the population/setting.

Furthermore, much of the existing research has focused on depression; widening this scope could enable policy and program responses that consider the potential range of mental health conditions related to inadequate food access. An emerging area of research is the link between food insecurity and disordered eating; in addition to the studies reviewed here focused on women, recent findings from a study of US adult men and women accessing a food pantry indicated a positive association between food insecurity and indicators of eating disorder pathology, such as binge eating and engaging in compensatory behaviors [96]. Additionally, few studies examined food security in relation to schizophrenia/psychosis or bipolar disorder among females.

The findings of the reviewed articles should be interpreted in light of several considerations. Most of the available research is based on US populations. While several studies were conducted among subpopulations such as women with children and African-American women, more research is needed to assess how food insecurity and mental health interact with other markers of vulnerability (such as single parenthood, insecure housing, drug use, experiences of violence, and immigrant/refugee status) in diverse subgroups. The majority of studies were cross-sectional, and causal inferences were not possible. Additionally, for longitudinal studies, in some cases, it was challenging to ascertain the timing of baseline and follow-up data collections. Adherence to checklists such as STROBE (Strengthening the Reporting of Observational Studies in Epidemiology) [97] could help promote transparency and accurate interpretation. Many authors noted limitations of self-reported data on mental health outcomes and food insecurity [16,17,26,29,30,31,32,34,40,44,55,62]. Some also noted temporal incongruence between measures of food insecurity and indicators of mental health [25,39,41] that may have affected their findings. Due to the varied emphases of the studies (including assessing feasibility and other characteristics of measures), a range of covariates and potential confounders were examined; in some cases, they were used to characterize samples whereas in others, they were included in statistical models such that it is difficult to compare estimates from one study to another. Finally, explicit approaches to account for the potential conceptual overlap between food insecurity and mental health indicators, such as feelings of worry or anxiety that are conceptualized as part of the experience of food insecurity and are also markers of psychological distress, were not common.

Considerations related to the review itself also warrant highlighting. We followed methodology for a scoping study [22] and, thus, did not conduct a formal appraisal of the quality of the included evidence, nor weight the evidence. Rather, our objective was to characterize the existing literature as to identify directions for future research. Further, although we employed a systematic search strategy and careful screening, our search was broad and it is possible that some relevant articles were inadvertently excluded. Additionally, we did not consider studies that presented pooled estimates for males and females. Although our interest was in females, this does not preclude the existence of associations between food insecurity and mental health among males, as observed in some reviewed studies that include stratified analyses. Additionally, given that we relied upon published articles, we did not account for publication bias in that research not supporting relationships between food insecurity and mental health may be less likely to have been identified.

## 5. Conclusions

Overall, this review supports a link between food insecurity and poor mental health among women in high-income countries. Despite gaps, the existing evidence is sufficient to warrant policy and program interventions to address these major public health challenges in a coordinated manner. An underlying theme of the literature is the complex ways in which food insecurity and mental health are connected both to each other and to an array of other issues, such as experiences of violence, housing circumstances, and life transitions such as pregnancy. These links underscore the need for coordinated approaches that consider how policy and program interventions can best address these complex issues and their interactions. Such approaches may be informed by systems methods [98,99,100] that consider the interplay among factors and how interventions to address one issue may affect another issue, influencing overall health and well-being.

Strategies to address financial inadequacy, such as a guaranteed basic income, have been called for to reduce vulnerability to food insecurity [19,101,102], and could play a role in ameliorating mental health conditions [103]. Additionally, food security screening has been recommended within clinical settings to enable referral to available community resources [13,104,105,106] (although it is imperative that practitioners have effective resources to which they can make referrals). While addressing the financial circumstances that underlie food insecurity is critical, screening for food access issues among those seeking treatment for mental health conditions could help build momentum in addressing the whole person instead of tackling issues in isolation, for example, helping health practitioners to understand, and potentially address, reasons for non-adherence to recommendations related to diet or other factors. Health and social service settings with integrated care models, in which women have access to a range of services that provide support during periods of food insecurity and poor mental health, may allow complex challenges to be addressed simultaneously [107]. In addition, health care providers are uniquely positioned to support individuals in accessing services such as government income-related benefits, dietary allowance benefits, or legal supports [16,106,108], and alongside individuals with lived experience of vulnerability, to advocate for increased financial supports and access to mental health care.

## Figures and Tables

**Table 1:** Overview of measures of food security drawn upon in articles (*n* = 39) examining associations between food insecurity and mental health among women in high-income countries.

Measure	Description	Abbreviated and Modified Versions	Articles Using Full Version	Articles Using Modified Versions
Community Childhood Hunger Identification Project	An 8-item scale developed by Wehler et al. [48]. Part of a survey instrument to examine the prevalence of hunger among low-income families. The items address qualitative and quantitative compromises among adults and children. Shown to have good specificity and sensitivity compared to evaluations of food security status based on household food inventories, dietary recall data, and other measures among a sample of women living with children in rural New York [49].	None	None	Wehler et al. 2004 [52]
Household Food Security Survey Module	An 18-item measure developed by the United States Department of Agriculture [8] and used to monitor household food security in the US and Canada. Measures the food security status of a household in the last 12 months. Items ask an adult respondent about anxiety related to the household food supply, running out of food, providing inadequately nutritious food, and substitutions or restrictions in food consumption by adults and/or children in the household due to lack of financial resources. Items are compiled to form a continuous, linear scale that categorizes households into one of four groups; food secure, marginal food secure, low food secure, and very low food secure [8]. Data from the HFSSM have been compared to household food expenditures and income [8] and dietary intakes [14], supporting its validity in capturing constrained food access due to inadequate financial resources.	Six-item short form: uses a subset of the 18-item survey. Does not characterize severe food insecurity and does not contain child-specific items. 10-item adult scale: includes only items referenced to adults in the household. Health Canada modifications: Refers to low food security as “moderate food insecurity” and very low food security as “severe food insecurity”. Less stringent than USDA coding, in that 2+ affirmative responses place an individual into a food insecure category.	Bronte-Tinkew et al. 2007 [53]; Casey et al. 2004 [54]; Chilton et al. 2013 [28]; Corman et al. 2016 [24]; Garg et al. 2015 [25]; Hanson et al. 2012 [15]; Hernandez et al. 2014 [26]; Huddleston-Casas et al. 2009 [55]; Laraia et al. 2006 [56]; Laraia et al. 2015 [27]; Lent et al. 2009 [45]; McCurdy et al. 2015 [29]; Sun et al. 2016 [30]; Trapp et al. 2015 [31] Health Canada coding: Muldoon et al. 2013 [32]; Tarasuk et al. 2013 [16]	Dressler et al. 2015 [37]; Kaiser et al. 2007 [57]; Laraia et al. 2009 [58]; Martin et al. 2016 [17] (Health Canada coding); Mathews et al. 2010 [34]; Peterman et al. 2013 [38]; Sharpe et al. 2016 [39]; Whitaker et al. 2006 [59]; 15-item adaptation for pregnant Latinas: Hromi-Fielder et al. 2011 [36] Other non-standard adaptations (3-, 4-, or 7-items): Ajrouch et al. 2010 [35]; Davey-Rothwell et al. 2014 [40]; Harrison et al. 2008 [60]; Melchior et al. 2009 [44]; Sidebottom et al. 2014 [33]
National Health and Nutrition Examination Survey-III (NHANES-III) food sufficiency indicators	NHANES-III was a health and nutrition survey conducted by the US Center for Disease Control (CDC). A food sufficiency component was included in the in-home adult questionnaire. Respondents were classified as “food insecure” if they “sometimes” or “often” did not have enough food to eat. Other questions included how many days in the prior month the respondent did not have money for food, reasons for not having enough food, and whether the respondent or child in the household had restricted their food intake due to lack of food [61].	None	Heflin et al. 2005 [62]; Siefert et al. 2007 [63]; Siefert et al. 2001 [64]	None
New Zealand measure of individual deprivation (NZiDep)	An 8-item scale measuring individual socioeconomic deprivation, specific to New Zealand. The scale has been validated among Maori, Pacific, and White New Zealand citizens [65]; criterion validity relied upon associations with tobacco smoking. Includes three-item composite measure of food security: “In the last 12 months have you personally made use of special food grants or food banks because you did not have enough money for food?” (yes/no), “In the last 12 months have you personally been forced to buy cheaper food so that you could pay for other things you needed?” (yes/no), “In the last 12 months have you personally gone without fresh fruit and vegetables often so that you could pay for other things you needed?” (yes/no).		Carter et al. 2011 [43]	None
Radimer–Cornell scale	A 12-item scale developed by Radimer et al. [47] at Cornell University based on qualitative research with low-income women. Twelve items cover aspects of household, adult, and child food insecurity. The content of the items address food anxiety, monotony of diet, financial constraints, food restriction, insufficient intake, and acquiring food in socially acceptable ways [47,66]. Shown to have good specificity and sensitivity compared to evaluations of food security status based on household food inventories, dietary recall data, and other measures among a sample of women living with children in rural New York [49]. Further information about the evolution of the instrument is available [67].	Single item	None	Sharkey et al. 2011 [41]
Other Multi- or Single-Item Measures			Birmingham et al. 2011 [42]; Klesges et al. 2001 [68]; Sharkey et al. 2003 [69]	None

**Table 2:** Overview of measures of mental health drawn upon in articles (*n* = 39) examining associations between food insecurity and mental health among women in high-income countries.

Measure	Description	Abbreviated Versions	Articles Using Full Version	Articles Using Abbreviated Versions
Center for Epidemiologic Studies, Depression Scale (CES-D)	A 20-item self-report scale measuring depressive symptoms in the general population. Components assess depressed mood, feelings of guilt and worthlessness, feelings of helplessness and hopelessness, psychomotor retardation, loss of appetite, and sleep disturbance in the prior week. Validity of the CES-D has been established through correlations with self-reported measures, clinical scores for depression, and other construct validity variables. Reliability and validity has been demonstrated across diverse characteristics of general population samples [71].	10-item short form 12-item short form	Ajrouch et al. 2010 [35]; Davey-Rothwell et al. 2014 [40]; Dressler et al. 2015 [37]; Hanson et al. 2012 [15]; Hromi-Fielder et al. 2011 [36]; Huddleston-Casas et al. 2009 [55]; Laraia et al. 2006 [56]; Laraia et al. 2009 [58]; Lent et al. 2009 [45]; McCurdy et al. 2015 [29]; Siefert et al. 2007 [63]	Bronte-Tinkew et al. 2007 [53]; Garg et al. 2015 [25]; Sharpe et al. 2016 [39]
Cohen’s Perceived Stress Scale (PSS)	A 14-item self-report Likert scale that measures the degree of unpredictability of the respondents’ life and the degree to which the respondent feels stress regarding these situations. Validated in young adult and post-secondary student population, the PSS correlated with physical and mental health related outcomes [77].	PSS-4 (4-item subset) 10-item short form	Laraia et al. 2006 [56]	Trapp et al. 2015 [31]; Laraia et al. 2015 [27]
Diagnostic Interview Schedule (DIS)	A structured interview designed for non-clinicians to assess and diagnose psychiatric disorders in respondents according to criteria in the Diagnostic and Statistical Manual of Mental Disorders, Fourth Edition (DSM-IV). The DIS has 19 diagnostic modules that cover different types of mental disorders. Within each module, respondents answer whether they have particular symptoms at the present, or have experienced them in the past [78].	None	Melchior et al. 2009 [44]	None
Edinburgh Postpartum Depression Scale (EPDS)	A 10-item self-report scale used to measure risk of postpartum depression in mothers within eight weeks of delivery. Items assess feelings of guilt, sleep deprivation, lack of energy, suicidality, and other general depressive symptoms experienced within the last 7 days. Validity has been examined in a sample of postpartum mothers, 6-weeks post-delivery, and compared with clinician diagnosis of depression [75].	3-item short form	None	Birmingham et al. 2011 [42]
Geriatric Depression Scale (GDS)	A 30-item self-report scale that assesses depression in geriatric populations (≥55 years). Items assess motivation, self-esteem, helplessness, mood, and agitation [76].	15-item short form	Klesges et al. 2001 [68]	Sharkey et al. 2003 [69]
Hopkins Symptom Checklist Subscale (HSCL)	A 58-item self-report scale used primarily with psychiatric outpatients, capturing five symptom dimensions including somatization, obsessive-compulsive, interpersonal sensitivity, depression, and anxiety [73]. Authors discuss a variety of studies in which the validity of the HSCL has been evaluated.	None	Klesges et al. 2001 [68]	None
Kemper 3-Item Screen	A 3-item self-report screening tool designed to assess maternal depressive symptoms. Validity examined with English-speaking mothers with children under 6 years of age, demonstrated 100% sensitivity and 88% specificity [74].	None	Casey et al. 2004 [54]; Chilton et al. 2013 [28]; Sun et al. 2016 [30]	None
Kessler-10 Scale	A 10-item screen developed for the US National Health Interview Survey. Designed to assess symptoms of general psychological distress through items on level of nervousness, hopelessness, lack of energy, depressive feelings, and worthlessness. Validity was examined with adults living in Australia, aged 18 years and older [79].	None	Carter et al. 2011 [43]	None
Patient Health Questionnaire (PHQ-9)	A 9-item questionnaire administered in a primary care setting by clinicians, designed to provide a diagnosis of major depressive disorder according to DSM guidelines. Items assess depressive symptoms and anhedonia experienced within the past 2 weeks. Validity was assessed among patients recruited through primary care offices, with 73% sensitivity and 94% specificity [80].	PHQ-2 (2-item subset)	Harrison et al. 2008 [60]; Sidebottom et al. 2014 [33]	Trapp et al. 2015 [31]
Pearlin’s Mastery Scale	A 7-item self-report Likert scale that measures the degree of control respondents feel they have over their lives. Authors note validation with individuals aged 18 to 65 years [81].	None	Heflin et al. 2005 [62]; Laraia et al. 2006 [56]	None
Rosenberg’s Self-Esteem Scale	A 10-item self-report Likert scale that assesses level of self-esteem in respondents [82].	None	Laraia et al. 2006 [56]; Laraia et al. 2009 [58]	None
SF-36 Health Survey	A 36-item health survey that consists of 5 physical health scales and 5 mental health scales. The mental component summary score is calculated from scores on 4 subscales; social functioning, role emotional, vitality, and mental health scales [83]. When tested on individuals 16–74 years of age, the SF-36 demonstrated good construct validity in patient population. Authors noted promise in use with the general population [83]	SF-12 (12-item short form)	Lent et al. 2009 [45]	Mathews et al. 2010 [34]
Spielberger’s Trait Anxiety Inventory	The Spielberger State-Trait Anxiety Inventory is a 20-item tool commonly used to measure anxiety, with higher scores indicating greater levels of anxiety [72]. The American Psychological Association has noted sensitivity of this inventory to predict distress overtime in caregivers [72].		Laraia et al. 2006 [56]	None
World Health Organization World Mental Health Composite International Diagnostic Interview (CIDI)	A comprehensive interview designed to diagnose major depressive disorder, other depressive disorders, anxiety disorders, substance abuse, and impulse control disorders according to the World Health Organization International Classification of Disease (ICD) and DSM criteria [70]. Evaluation studies suggested good test-retest and interrater reliability, and its use in different settings and countries was deemed acceptable [84].	CIDI short form (CIDI-SF), also referred to as screening version	None	Corman et al. 2016 [24]; Heflin et al. 2005 [62]; Hernandez et al. 2014 [26]; Martin et al. 2016 [17]; Siefert et al. 2001 [64]; Whitaker et al. 2006 [59]

## Appendix A

**Table A1:** Key characteristics of articles (*n =* 39 from 31 studies/surveys) included in scoping review of food insecurity and mental health among women in high-income countries, by measure of food insecurity.

Reference	Sample (Participants (Age), Setting, Race/Ethnicity, Data Source)	Study Design (Sample Size)	Purpose	Food Security Measure	Mental Health Measure	Mental Health States/Conditions	Covariates Considered	Analytic Approach and Key Findings
**Household Food Security Survey Module (HFSSM)**								
*Longitudinal analyses*								
Bronte-Tinkew et al. 2007 [53]	**Mothers** (mean age, 27.5 years), US, race/ethnicity not specified Early Childhood Longitudinal Study-Birth Cohort (ECLS-B)	Longitudinal (8693)	Examine association between food insecurity and child health, and examine parental depression and behaviors as mediators	USDA HFSSM	CES-D 12-item subset Authors note strong psychometric properties	Symptoms of maternal depression	Parent education, maternal employment, maternal age (at birth), family structure, receipt of food subsidy, child exposure to cigarette smoke, number of well-baby visits, household poverty index ratio	Structural equation modeling: Mothers in food-insecure households reported higher levels of depressive symptoms (β = 0.243, *p* < 0.001), which were associated parent-reported fair or poor health in children at 24 months.
Corman et al. 2016 [24]	**Mothers** (mean age, 25 years) from 75 birth hospitals in 20 US cities, included White, African American, Hispanic, and other races/ethnicities Fragile Families and Child Wellbeing Study	Longitudinal (2965)	Examine association between maternal depression in the postpartum year, housing conditions, and food insecurity	USDA HFSSM	CIDI short form Authors note that the measure has been validated	Clinical diagnosis of a MDE (defined as 3+ symptoms of dysphoria or anhedonia for most of the day for a period of at least 2 weeks) during the postpartum year (assessed at 1 year)	Maternal, paternal, and prenatal housing characteristics (measured at baseline), maternal grandparents‘ mental illness and child characteristics	Multivariable analysis: Compared to women who did not report depression, mothers who reported depression were more likely to experience inadequate housing at 2–3 years due to lack of heat (aOR 1.57, 95% CI 1.11–2.22) and energy insecurity (aOR 1.69, 95% CI 1.24–2.30). Depression was associated with combinations of hardships, including inadequate housing, housing instability, and food insecurity (aOR 3.85, 95% CI 1.34–11.11).
Garg et al. 2015 [25]	Low-income **mothers** (mean age, 25 years) and their young children in the US, non-Hispanic White, non-Hispanic Black, Hispanic, Asian-Pacific Islander, other races/ethnicities Early Childhood Longitudinal Study, Birth Cohort (ECLS-B)	Longitudinal (2917)	To determine impact of maternal depression on future household food insecurity in low-income households with young children.	USDA HFSSM	CES-D 12 item Authors note that the short form has been previously validated.	Depressive symptoms	Maternal and household characteristics including race/ethnicity, age, marital status, employment, education, mothers’ foreign-born status, household income, and maternal self-reported health status.	Multivariable analyses: Maternal depression at baseline (9 months) was associated with food insecurity at follow-up (24 months) (aOR 1.50, 95% CI 1.06–2.12). Mothers who reported depressive symptoms and received WIC at baseline were more likely (aOR 1.59, 95% CI 1.15–2.21) to experience food insecurity at follow-up.
Hanson et al. 2012 [15]	Low income, rural **mothers** (mean age, 30 years) in US, White and non-White races/ethnicities Rural Low-Income Families: Monitoring Their Well-being and Functioning in the Context of Welfare Reform	Longitudinal (225)	Examine food insecurity and various risk factors, including human capital, social support, and financial situation, among rural low-income families with children.	USDA HFSSM	CES-D 20-item	Depressive symptoms	Education, 3 or more chronic health conditions, food and financial skills, high support for parenting, home ownership at baseline, employment, housing assistance, participation in SNAP assistance, health insurance	Multivariable analyses: Compared to women having no years at risk for depression, women classified as at risk for depression for 2 consecutive years had 4.28 times greater odds of experiencing persistent versus no food insecurity (*p* < 0.01), and 3.65 times greater odds to experience persistent versus discontinuous food insecurity (*p* < 0.05).
Hernandez et al. 2014 [26]	Low-income, urban, unmarried **mothers** (mean age, 28 years) of newborn children recruited from 75 birth hospitals in 20 US cities, White, African American, Hispanic, and other races/ethnicities Fragile Families and Child Well-being Study	Longitudinal (1690)	Examine association between intimate partner violence, depression, and household food insecurity	USDA HFSSM	CIDI short form	Clinical diagnosis of depression; depressive symptoms	Mothers’ age, race/ethnicity, education, employment, relationship status, household income, number of children, baseline food security	Multivariable analyses: Mothers reporting depression were twice as likely to be food-insecure two years later compared to mothers who did not report depression (aOR 2.03, 95% CI 1.45–2.84). The relationship between intimate partner violence and food insecurity among women was mediated by depression (z = 2.89, *p* < 0.01).
Lent et al. 2009 [45]	Rural, low-income **mothers** (18+ years), recruited through local educators, WIC and Even Start programs in New York, US, majority White. Rural Families Speak: Tracking the Well-Being and Functioning of Rural Families in the Context of Welfare Policies Study	Longitudinal (mixed methods) (29)	Examine the temporal/causal relationship and potential mechanisms between mental health conditions such as depression and household food insecurity	USDA HFSSM	CES-D 20-item, SF-36 Health Survey (mental health scales: Vitality, Social Functioning, Role Emotional, Mental Health)	Depressive symptoms	Not applicable	Unadjusted analyses: High levels of depressive symptoms (according to the CES-D) at wave 2 were correlated with remaining food-insecure at wave 3 (*p* = 0.009); reverse relationship not significant. Unhealthy scores on the mental health scores at wave 2 were also associated with remaining food-insecure at wave 3 (*p* = 0.01). Qualitative analyses suggest that poor mental health contributes to persistence of food insecurity by limiting employment.
Huddleston-Casas et al. 2009 [55]	Rural **mothers** (mean age, 30 years) recruited from programs serving low-income populations in 17 US states, included White, African American, Latina, and other races/ethnicities NC-223, Rural Families Speak Study	Longitudinal (413)	Examine direction of the relationship between household food insecurity and depression over three annual waves of data	USDA HFSSM	CES-D 20-item Authors note that reliability in this sample matched that documented for the general population	Depressive symptoms	Age, ethnicity, household income, marital status, education	Structural equation modeling (using data for 413 women, with sensitivity analysis with 184 women who had depression data for three waves): A bidirectional relationship between food insecurity and depression (X^2^/df = 1.835, RMSEA = 0.068, CFI = 0.989) was observed.
Laraia et al. 2015 [27]	**Pregnant women** (16+ years), US, included White, Black, other races/ethnicities Pregnancy, Infection, and Nutrition (PIN) Postpartum study, recruited from University of North Carolina Hospitals and private clinics	Longitudinal (526)	To examine relationship between food insecurity and perceived stress, disordered eating, dietary intake, and postpartum weight status	USDA HFSSM, 18 items (between 27 and 30 weeks’ gestation) and 6-item short form (12 months postpartum)	Cohen’s Perceived Stress Scale (PSS) 10-item, Eating Attitude Test (EAT) 26 item Authors note that Cohen’s Perceived Stress has been validated in pregnant women	Perceived stress, disordered eating	Maternal race, age, marital status, education, parity, physical activity, smoking during pregnancy and postpartum, breastfeeding postpartum, poverty level	Multivariable analyses: Women living in food-insecure households during pregnancy had higher levels of perceived stress (β = 3.36, 95% CI 0.79–5.92) and higher scores for disordered eating (β = 1.95, 95% CI 0.25–4.16) at 3 months postpartum and higher levels of perceived stress (β = 3.67, 95% CI 0.94–6.41) at 12 months postpartum compared to those living in food-secure households during pregnancy. Women who experienced any level of household food insecurity during the postpartum period had higher perceived stress (β = 6.12, 95% CI 3.86–8.38), and higher scores for disordered eating (β = 1.79, 95% CI 0.03–3.62) compared to women in food-secure households.
*Cross-sectional analyses*								
Casey et al. 2004 [54]	Female **caregivers** (age not specified), US, women of African American, White, and Hispanic race/ethnicity Children’s Sentinel Nutrition Assessment Program (C-SNAP), recruited from medical centers in several large US cities	Cross-sectional (5306)	Examine nature of the relationship between depression, food insecurity, and loss of social assistance and its impact on child health	USDA HFSSM	Kemper 3-item screen Authors note sensitivity of 100%, specificity of 88%, and positive predictive value of 66% compared to an 8-item screening instrument	Maternal depressive symptoms	Study site location, race, insurance type, education, and low birth weight	Multivariable analysis: Mothers experiencing food insecurity had greater odds of positive depression screen compared to those from food secure households (aOR 2.69, 95% CI 2.33–3.11). Mothers experiencing a decrease or sanction in food stamp status had increased odds of reporting a positive depression screen, compared to those with no decrease in food stamp status (aOR 1.26, 95% CI 0.97–1.65 and aOR 1.56 95% CI 1.06–2.30, respectively).
Chilton et al. 2013 [28]	**Mothers** (mean age, 26.7 years) in Philadelphia, US, African American, White, Hispanic races/ethnicities Recruited from public assistance programs through the Children’s Health Watch study	Cross-sectional (mixed methods) (44)	Explore aspects of exposure to violence related to food insecurity among lone mother households.	USDA HFSSM	Kemper 3-item screen	Maternal depressive symptoms	Not applicable	Descriptive estimates: A higher proportion of mothers living with very low food security reported depressive symptoms (71%) compared to those with low food security (53%) and food-secure (17%) mothers. Women living with very low food security (53%) were more likely to have experienced life-changing violence in childhood compared to those with low food security (33%) and food secure (33%) mothers.
McCurdy et al. 2015 [29]	Low-income **mothers** (mean age, 30.1 years) and children recruited from 7 preschools in low-income urban neighborhoods in the US, included Hispanic and non-Hispanic races/ethnicities	Cross-sectional (166)	To determine correlates of weight, including food security, among low-income, ethnically diverse mothers and examine role of mental health	USDA HFSSM	CES-D 20-item Authors note high internal consistency for the measure and note acceptable internal reliability in this sample	Depressive symptoms	Not applicable	Bivariate analyses: Mothers living in food-insecure households had more depressive symptoms compared to food-secure mothers (t = 2.26, *p* < 0.02).
Sun et al. 2016 [30]	**Mothers** (mean age, 24 years) of young children (aged < 4 years), US, non-Hispanic White, non-Hispanic Black, Hispanic, other races/ethnicities, recruited from Philadelphia hospitals.	Cross-sectional (1255)	To examine association between adverse childhood experiences among mothers and household and child food insecurity determine associations with depressive symptoms	USDA HFSSM	Kemper 3-Item Screen, ACEs scale for Adverse Childhood Experiences Authors note that the Kemper 3-item is validated as a proxy for a longer screener with 100% sensitivity, 88% specificity, and 66% positive predictive value, and ACEs scale has been validated and shown to have good test-retest reliability.	Depressive symptoms, adverse childhood experiences, such as abuse, neglect, and household dysfunction	Caregiver’s age and self-rated health, caregiver’s participation in nutrition programs, race/ethnicity, marital status, employment, education, and child’s health insurance,	Depressive symptoms were reported among 18.4% of women in food-secure households, 48.6% of those in households with low food security, and 54.4% of those in households with very low food security (*p* < 0.01). Multivariable analyses: Mothers who reported depressive symptoms and 4+ adverse childhood experiences were 2.3 times (95% CI 1.0–5.3) as likely to report low food security, 6.6 times (95% CI 2.1–20.5) as likely to report indications of very low food security compared to those reporting depressive symptoms but no adverse childhood experiences. In addition, mothers who reported depressive symptoms and 4+ adverse childhood experiences were 17.6 times (95% CI 7.3–42.6) as likely to report child food insecurity compared to those who reported no depressive symptoms and no adverse childhood experiences.
Trapp et al. 2015 [31]	Low-income children (2–4 years) and **mothers** (18+ years), US, Hispanic, African-American races/ethnicities Steps to Growing Up Health study, primary care-based intervention	Cross-sectional (222)	Examine relationship between food security, diet, and weight status among urban preschool children, and examine whether maternal depression and stress acts as a mediator	USDA HFSSM	PHQ-2, Cohen’s Perceived Stress Scale 4-item subset (PSS-4) Authors note that the PHQ-2 has good validity, and identified the sensitivity and specificity of the cutoff used for risk for major depression	Depressive symptoms and perceived stress	Household size, primary home language, marital status, employment, household income	Bivariate analyses: Mothers living in food-insecure households were more likely to report depressive symptoms compared to food-secure mothers (27% vs. 9%; *p* < 0.001), but perceived stress scores were not different between food-insecure and food-secure mothers (*p* = 0.5).
Laraia et al. 2006 [56]	Low-income **pregnant women** (mean age, 29 years), US, included African American, White, and other races/ethnicities Pregnancy, Infection, and Nutrition (PIN) cohort study, recruited from University of North Carolina Hospitals and private clinics	Cross-sectional (606)	Examine prevalence and determinants of food insecurity among pregnant women from medium- and low-income women	USDA HFSSM	Cohen’s Perceived Stress Scale 14-item, Spielberger’s Trait Anxiety Inventory 20-item, CES-D 20-item, Rosenberg’s Self Esteem Scale 10-item, Pearlin’s Mastery Scale 7-item, Levenson’s IPC Locus of Control 24-item Authors note stability and internal consistency of measures	Perceived stress, anxiety, depressive symptoms, self-esteem, mastery, locus of control	Mother’s age, number of children, household income, education, race, marital status	Multivariable analyses: Perceived stress (aOR 2.24, 95% CI 1.63–3.08), trait anxiety (aOR 2.14, 95% CI 1.55–2.96), depressive symptoms (aOR 1.87, 95% CI 1.40–2.51), and feeling that ones’ destiny is up to chance (aOR 1.67, 95% CI 1.20–2.32) were positively associated with household food insecurity. Women living in food-insecure households were less likely to report feelings of mastery over their lives (aOR 0.49, 95% CI 0.35–0.68) and high self-esteem (aOR 0.52, 95% CI 0.38–0.69).
Muldoon et al. 2013 [32]	Adults (18–64 years), Canada 2007–2008 Canadian Community Health Survey	Cross-sectional (sample subset of 5588 reporting indications of food insecurity in the past year)	Examine rates of mental illness among Canadian adults who lived in food-insecure households with and without hunger	USDA HFSSM (Health Canada coding)	Self-reported diagnosis of chronic health conditions diagnosed by a health professional	Clinical diagnoses of mood or anxiety disorders	Education, age, single parent household status, immigrant status	Multivariable analyses: Females experiencing food insecurity with hunger had greater odds (aOR 1.89, 95% CI 1.62–2.20) of reporting a depression diagnosis compared to women who did not report food insecurity with hunger.
Tarasuk et al. 2013 [16]	Adults (18–64 years), Canada 2007–2008 Canadian Community Health Survey	Cross-sectional (77,053)	Examine whether chronic physical and mental conditions health conditions are associated with household food insecurity	USDA HFSSM (Health Canada coding)	Self-reported presence of chronic health conditions diagnosed by a health professional	Clinical diagnoses of mood or anxiety disorders	Age, sex, province, education, household type, median household income, main source of household income, and home ownership	Multivariable analysis: Self-reported diagnoses of 3 or more chronic physical and mental health conditions raised the odds of a woman experiencing severe food insecurity (aOR 2.15, 95% CI 1.50–3.10) compared to fewer or no chronic conditions Among women in food-secure households, 11.6% reported mood or anxiety disorders; among those in marginally food-secure, moderately food-insecure, and severely food-insecure households, the prevalences were 20.3%, 26.8%, and 47.1%, respectively.
**Abbreviated/adapted versions of Household Food Security Survey Module (HFSSM)**								
*Longitudinal analyses*								
Melchior et al. 2009 [44]	**Mothers** of twins (average 35.5 years) from England and Wales, Britain, included White and non-White races/ethnicities. Environmental Risk Study	Longitudinal (1116)	Examine the association between food insecurity and maternal depression, psychosis spectrum disorder, alcohol or drug abuse, and intimate partner violence	USDA HFSSM, 7-item short form	Diagnostic Interview Schedule (DIS)	Depressive symptoms, psychotic symptoms	Mother’s age, income, ethnicity, marital status, household size, mother’s employment, mother’s reading ability	Multivariable analyses: Food insecurity increased the odds of depression (OR 2.12, 95% CI 1.61–4.93), intimate partner violence (OR 2.36, 95% CI 1.18–4.73), and psychosis (OR 4.01, 95% CI 2.03–7.94) among women two years later. Food insecurity was associated with mental illness comorbidity in mothers—29% of food-insecure mothers had experienced mental health problems or intimate partner violence.
Sidebottom et al. 2014 [33]	**Pregnant women** (mean age, 22 years) recruited from Health Centres in Minneapolis and St. Paul, US, included African American, American Indian, Asian/Pacific Islander, Hispanic (any race), White, and bi/multiracial women Data from the Twin Cities Healthy Start Program	Longitudinal (prenatal and postpartum assessments) (594)	Examine correlates of depression in pregnancy and postpartum period	USDA HFSSM, 4-item subset	PHQ-9 with modification of the item measuring psychomotor issues (split into 2 questions but scored as one) Authors noted sensitivity of 77%, specificity of 94%, and positive predictive value of 59% in primary care populations, with higher values in populations with a high prevalence of depressive disorder	Depressive symptoms	Age, race/ethnicity, foreign-born, lack of social support, abuse of any kind, child protection involvement, living with child’s father, drug, alcohol and cigarette use, lack of phone access, and housing instability	Multivariable analyses: Compared to women who had low depressive symptom levels in both the prenatal and postpartum periods, the odds of elevated depressive symptoms prenatally were higher (aOR 2.44, 95% CI 1.43–4.16) among those with low levels of food security. Food security and depressive symptoms in the postpartum period were not related.
*Cross-sectional analyses*								
Whitaker et al. 2006 [59]	**Mothers** (18+ years) of 3-year old children, recruited from 75 birth hospitals in 20 US cities. Included White, African American, Hispanic, other races/ethnicities Fragile Families and Child Wellbeing Study	Cross-sectional (2870)	Examine if food security is associated with prevalence of depression and anxiety in mothers and behavior problems in children	USDA HFSSM, 10 adult-referenced items	CIDI short form administered 3 years after child’s birth, modified cut-off for a major depressive episode (MDE) based on symptoms of anhedonia	Clinical diagnosis of a MDE or generalized anxiety disorder (GAD) in the prior 12 months	Mother’s education, race/ethnicity, relationship status, employment in previous year, binge drinking, illicit drug use, global health, prenatal smoking, prenatal physical domestic violence, household income/poverty ratio, number of children, non-food related material hardship, and whether father was ever in jail	Multivariable analyses: Compared to fully food-secure mothers, experiencing marginal food insecurity increased the odds of experiencing an MDE or GAD (aOR 1.4, 95% CI 1.1–1.8; and aOR 1.7, 95% CI 1.0–2.7, respectively). Compared to fully food-secure mothers, experiencing food insecurity increased the odds of experiencing an MDE or GAD (aOR 2.2, 95% CI 1.6–2.9; and aOR 2.3, 95% CI 1.5–3.6 respectively). Mothers experiencing food insecurity twice as likely to also experience either MDE or GAD compared to food-secure mothers (aOR 2.2, 95% CI 1.6–2.9).
Laraia et al. 2009 [58]	African American, first-time **mothers** (18–35 years) recruited from Special Supplemental Nutrition Program for Women, Children, and Infants (WIC) clinics in North Carolina, US Infant Care, Feeding, and Risk of Obesity observational study	Cross-sectional analysis of longitudinal study, focused on 3-month postpartum baseline data (206)	Identify maternal and household correlates of food insecurity among African-American mothers	USDA HFSSM, 6-item short form	CES-D, Rosenberg Self-Esteem Scale	Depressive symptoms and self-esteem	Maternal age, education, work status, depression score, and self-esteem, as well as household composition (presence of father, grandmother and household size)	Bivariate analyses: Women living in food-insecure households had significantly higher scores on the depressive scale compared to food-secure women (*p* < 0.05). Multivariable analyses: Depressive symptoms were associated with marginal food security and food insecurity (aRRR * 1.04, 95% CI 1.00–1.08 and aRRR * 1.10, 95% CI 1.04–1.16, respectively). Self-esteem scores were negatively associated with risk for marginal food security and food insecurity (aRRR * 0.91, 95% CI 0.84–0.98, and aRRR * 0.89, 95% CI 0.79–0.99, respectively) * aRRR = adjusted Relative Risk Ratio.
Mathews et al. 2010 [34]	**Mothers** (<25 years) recruited from the Special Supplemental Nutrition Program for Women, Infants, and Children (WIC) clinics in Butte County, California, US, included White, non-White races/ethnicities	Cross-sectional (155)	Evaluate the prevalence of and associations between food insecurity and health status among women participating in WIC	USDA HFSSM, 6-item short form	SF-12 Health Survey Authors noted that the SF-12 has been validated previously	General mental (and physical) health symptoms	Diet choice score, income, ethnicity, age, education	Bivariate analyses: Women experiencing low or very low food insecurity had significantly lower mental health scores, indicating more mental health symptoms compared to food-secure women (*p* < 0.001). The correlation between food insecurity and mental health scores indicates that as women’s food security increased, mental health also increases. Multivariable analyses: The likelihood of having a good mental health score was lower (OR 0.41, 95% CI 0.16–0.73) among those in food-insecure versus those in food-secure households.
Ajrouch et al. 2010 [35]	Female African-American **caregivers** (mean age, 30.8 years) of young children recruited from high-poverty census tracts in Detroit, US Detroit Centre for Research on Oral Health Disparities.	Cross-sectional (multiple waves of data collection, relevant variables were assessed in wave 2) (736)	Explore link between situational stressors, including food insufficiency, and psychological distress, and examine social support as a potential mediator	USDA HFSSM, 3-item subset (referred to as food insufficiency) Cronbach’s alpha reported as 0.79	CES-D 20-item Authors noted high internal reliability in this sample	Depressive symptoms	Age, self-rated health, and education level	Multivariable analyses: Higher food insufficiency associated with higher depressive symptoms (referred to as psychological distress) (β = 2.88, *p* < 0.001). At high levels of stress, social support was not a mediator of this relationship.
Hromi-Fielder et al. 2011 [36]	Low income, **pregnant Latina women** (mean age, 25 years), recruited from local agencies and programs in Hartford, Connecticut, US.	Cross-sectional (135)	Assess relationship between household food insecurity and prenatal depressive symptoms	USDA HFSSM, 15-item subset adapted version for pregnant Latinas Authors note that the adapted version was validated for this population	CES-D 20-item Authors note that the CES-D has been validated with multi-ethnic samples, including Mexican-Americans	Prenatal depressive symptoms	Parity, heartburn during pregnancy, self-reported health during pregnancy, history of depression, Latina subgroup, acculturation	Multivariable analyses: Women experiencing food insecurity were more likely to report high levels of prenatal depressive symptoms compared to those who were food secure (aOR 2.59, 95% CI 1.03–6.52).
Harrison et al. 2008 [60]	**Pregnant women** in Minneapolis and St. Paul, US, included African American, Asian/Pacific Islander, Hispanic, American Indian, White, bi/multiracial races/ethnicities. recruited from Federally Qualified Health Centres Feasibility study associated with Twin Cities Healthy Start Program	Cross-sectional (1386)	Examine the prevalence, co-occurrence, and inter-correlations of self-reported psychosocial risk factors, including food insecurity.	USDA HFSSM, 4-item subset	PHQ-9, intimate partner violence items, 8 items from the Maternal Social Support Index Authors note high levels of internal reliability, test-retest reliability, sensitivity, and specificity for PHQ-9	Depressive symptoms	Not applicable	Bivariate analyses: Depressive symptoms (r = 0.267), social support (r = 0.194), and intimate partner violence (r = 0.173) were significantly correlated (*p* ≤ 0.0001) with household food insecurity.
Martin et al. 2016 [17]	Adults (18–75 years), Canada Data from the 2009–2010 Canadian Community Health Survey	Cross-sectional (100,401)	To examine the co-occurrence of food insecurity and mental illness across varying levels of stress and community belonging	USDA HFSSM, 10 adult-referenced items (Health Canada coding)	Self-reported diagnosis of a mood or anxiety disorder, subsample (*n* = 47,942) completed CIDI short form, one item for each of perceived stress and community belonging	Clinical diagnosis of a mood disorder such as depression, bipolar disorder, mania, or dysthymia; or an anxiety disorder such as phobia, obsessive-compulsive disorder, or panic disorder. Past 12 months of major depression from CIDI short form.	Age, marital status, children in house, household income, education, unemployment, and self-perceived physical health, as well as overall stress level and community belonging.	Multivariable analyses: Women living in severely food-insecure households had 18.4% (95% CI 16.7–20.1) greater adjusted prevalence of a mental disorder compared to those living in food-secure households. The prevalence of women reporting high levels of stress increased with worsening food security. Greater proportions of severely food-insecure women reported low community belonging compared to more food-secure women. Interaction between community belonging, food insecurity, and perceived stress not significant.
Dressler et al. 2015 [37]	Low-income women (18–64 years) recruited from homeless shelters, food pantries, libraries, soup kitchens, and community centers, US, included African American, White, Native American women	Cross-sectional (330)	Examine depression and its relationship with food insecurity, weight status, emotional eating, and dietary intake among low-income women	USDA HFSSM, 6-item short form	CES-D 20-item, emotional eating questions developed using validated questionnaires Authors note that the CES-D is valid and reliable and note the internal consistency in the sample for both the CES-D and the emotional eating questions	Symptoms of depression and emotional eating	Not applicable	Bivariate analyses: Women categorized as depressed had higher food insecurity scores compared to women who were not depressed (3.2 vs. 1.9, *p* < 0.05). Depression and emotional eating were also associated.
Kaiser et al. 2007 [57]	Women (18+ years) living in California, US, included White, African American, Hispanic/Latino, and other races/ethnicities. 2004 California Women’s Health Survey	Cross-sectional (4037)	Identify factors associated with food insecurity	USDA HFSSM, 6-item subset, modified to refer to respondent and not to other adults in household	Indicators of mental or emotional problems	Mental, (physical), or emotional problems that interfere with daily life, feeling depressed or sad, and feeling overwhelmed	Income as a proportion of the federal poverty ratio	Multivariable analyses: Higher food insecurity was associated with feeling depressed or sad for 2+ days in the prior month (aOR 1.61, 95% CI 1.28–2.02), feeling overwhelmed in past 30 days (aOR 3.10, 95% CI 2.49–3.85), and reporting that physical or mental health conditions interfered with normal activities in past 30 days (aOR 1.81, 95% CI 1.45–2.27).
Peterman et al. 2013 [38]	Cambodian women (30–65 years) recruited from clients of the Cambodian Mutual Assurance Association of Lowell, Massachusetts, US Cambodian Community Health Program 2010	Cross-sectional (150)	Examine post-immigration experiences with food, food security status, and correlates among refugee women	USDA HFSSM, 6-item short form	Harvard Program in Refugee Trauma’s depression scale; 14 items, previously translated and validated for use in Cambodian refugee populations	Clinical diagnosis of depression	Marital status, receipt of food stamps, income to poverty ratio, acculturation, age	Multivariable analyses: Women experiencing marginal/low/very low food security were more likely (aOR 3.73, 95% CI 1.26–11.05) to be classified as depressed compared to those in food-secure households.
Sharpe et al. 2016 [39]	Low-income women (25–51 years) recruited from 18 census tracts in which 25% or more of residents had below-poverty income in South Carolina, US, mainly African-American Sisters Taking Action for Real Success (STARS) trial	Cross-sectional (202)	Examine whether on diet quality and psychosocial and behavioral factors are associated with household food security	USDA HFSSM, 6-item short form	CES-D 10 item, emotional eating subscale of the Eating Behavior Patterns Questionnaire Authors noted that the CESD-10 has been validated and that the Eating Behavior Patterns Questionnaire has been shown to have acceptable internal consistency and construct validity in African-American women; authors also identified Cronbach’s alphas for both measures in the study sample	Symptoms of depression and emotional eating	Not applicable	Bivariate analyses: Women experiencing food insecurity had significantly higher scores for depressive symptoms (indicating more symptoms) compared to women living in food-secure households (mean score 10.9 (SD 6.1) vs. 8.3 (SD 5.0), t = 3.36, *p* < 0.001). Women experiencing food insecurity had significantly lower emotional eating scores (indicating higher levels of emotional eating) compared to women living in food-secure households (mean score 10.2 (SD 3.1) vs. 11.4 (SD 3.8), t = 2.45, *p* < 0.02).
Davey-Rothwell et al. 2014 [40]	Low-income women (18–55 years) at risk for HIV, recruited through street outreach and public advertisements in the US, majority African-American women Data from the CHAT study	Cross-sectional (based on 6-month visit) (443)	Explore food insecurity among drug-using and non-drug-using women and examine the relationship between depression and food insecurity	USDA HFSSM, 4-item subset Authors noted acceptable internal consistency in this sample	CES-D 20-item Authors noted high internal consistency in this sample	Depressive symptoms	Age, race, income, receipt of food stamps	Multivariable analyses: Drug-users were 2.71 times (aOR, 95% CI 1.51–4.88), and non-drug-users were 5.9 times (aOR, 95% CI 2.80–12.45) more likely to experience depression if they were food insecure compared to food secure.
**Radimer–Cornell Scale**								
*Cross-sectional analyses*								
Sharkey et al. 2011 [41]	Urban and rural women (18+ years) living in Brazos Valley, Texas, US, included White and non-White races/ethnicities Brazos Valley Health Status Assessment	Cross-sectional (1367)	Examine health status, mental distress, and household food insecurity among urban and rural women	Radimer–Cornell Scale, first item focused on food deprivation (food we bought did not last and we did not have enough money to buy more) was used to determine presence of household food insecurity Authors noted that the Scale has been shown to be valid for non-white participants	Centre for Disease Control (CDC) and the Behavioral Risk Factor Surveillance Systems (BRFSS) questionnaire to assess health-related quality of life (perceived mental—and general and physical—well-being—thinking about your mental health, which includes stress, depression, and problems with emotions, for how many days during the past 30 days was your mental health not good?) Authors noted that the measures have been shown to be valid for non-white populations	Perceived mental health (stress, depression, problems with emotions), referred to as frequent mental distress	Age, race, education, annual household income, employment, rural vs. urban geographic location	Multivariable analyses: Women experiencing food insecurity in the last 30 days were more likely to frequently experience mental distress compared to food-secure women (aOR 2.25, 95% CI 1.59–3.18).
**Community Childhood Hunger Identification Project (CCHIP) measure**								
*Cross-sectional analyses*								
Wehler et al. 2004 [52]	Homeless and housed women (mean age, 28 years) recruited from Worcester’s homeless shelters and welfare hostels and the Department of Public Welfare office, US, included White, African American, Hispanic, and other races/ethnicities. Worcester Family Research Project	Cross-sectional (354)	Examine factors associated with adult or child hunger among low-income housed and homeless female-headed families	CCHIP, 7 items querying adult and child hunger Authors noted high level of internal consistency and factor analysis indicated a single-factor solution	Structured Clinical Interview for Diagnostic Statistical Manual (DSM-III-R non-patient edition), Life Experiences Survey	Clinical diagnosis of substance use, depression, posttraumatic stress disorder (PTSD), major life events in adulthood (e.g., violence)	Age, ethnicity, housing status, marital status, acculturation, parenting status, parent substance abuse, foster care status, number and age of children, income, psychological factors (coping and parental hassles), social service utilization, social network size	Exploratory analytic approach identified factors differentiating families with child hunger from those with no hunger, these did not include the mental health factors. Multivariable analyses: The experience of sexual abuse in childhood increased the odds of adult hunger (aOR: 4.23, 95% CI 2.28–7.82); intimate partner violence in adulthood and a PTSD diagnosis appeared to be mediators of the childhood sexual abuse-current hunger association. Financial support from a sibling reduced the odds of experiencing food insecurity.
**NHANES-III food insufficiency indicators**								
*Longitudinal analyses*								
Heflin et al. 2005 [62]	**Mothers** (18–54 years) receiving public assistance in urban Michigan, US, included African American, non-Hispanic White races/ethnicities Women’s Employment Study	Longitudinal (753)	Examine effect of food insecurity on the mental health status of welfare recipients over a 3-year period	NHANES-III food insufficiency question Authors noted that this measure is widely accepted as a valid measure of food insufficiency	CIDI short form, Pearlin Mastery Scale 7 item	Clinical diagnosis of depression, mastery (degree to which individuals perceive themselves to be in control of their own lives)	Household size, marital status, household income, poverty-related stressful life circumstances, neighborhood hazards, domestic violence, experiences of discrimination based on race and gender	Multivariable fixed effects models: Changes in food insecurity significantly predict changes in major depression status after adjusting for changes in household composition and socio-environmental stressors (β = 0.75, SE 0.24. *p* < 0.01). No association observed between changes in food insufficiency status and changes in mastery.
*Cross-sectional analyses*								
Siefert et al. 2001 [64]	Single women receiving welfare (mean age, 28 years) living in urban Michigan, US, included African-American, White women Women’s Employment Study	Cross-sectional (724)	Examine relationship between food insufficiency and physical and mental health among low-income women	NHANES-III food insufficiency question Authors noted that this measure is widely accepted as a valid measure of food insufficiency	CIDI short form Authors noted that acceptable test-retest reliability and clinical validity have been observed	Clinical diagnosis of major depressive disorder and generalized anxiety disorder	Self-rated health, physical limitations, age, number of children in the household, education level, poverty level, employment, poverty-related stressful life events and conditions	Multivariable analyses: Food insufficiency significantly predicted major depressive disorder (aOR 2.21, 95% CI 1.48–3.29). The association between food insufficiency and generalized anxiety disorder, adjusted for covariates, was not significant.
Siefert et al. 2007 [63]	African-American **mothers** (mean age, 28 years) recruited from 39 high-poverty census areas in Detroit, US Detroit Center for Research on Oral Health Disparities	Cross-sectional (multiple waves of data collection, relevant variables were assessed in wave 1) (824)	Determine correlates of depressive symptoms among low-income mothers	NHANES-III food insufficiency questionAuthors noted that this measure is widely accepted as a valid measure of food insufficiency	CES-D, 20-item Authors noted that the CES-D is a reliable and well-validated sale, with standard scoring widely used in research, four-factor structure found in the general population has also been found in African-Americans with low socioeconomic status	Depressive symptoms	Living in poorly maintained housing, not being employed, experiences of everyday discrimination, instrumental and emotional social support, age, education, household size, number of children <18 years of age, income	Bivariate analyses: Mothers with depressive symptoms more likely to report household food insufficiency (14.5%) compared to women without depressive symptoms (6%). Multivariable analyses: In models adjusted for income and education, living in a food-insufficient household was associated with 2.5 greater odds (95% CI 1.25–4.98) of maternal depressive symptoms. Instrumental social support was a protective factor.
**Other brief measures**								
*Cross-sectional analyses*								
Birmingham et al. 2011 [42]	**Mothers** of newborns (mean age, 25 years), US., included African American, Hispanic, White, Asian, other races/ethnicities, recruited from urban pediatric emergency departments	Cross-sectional (195)	To examine the performance of the Edinburgh Postpartum Depression Scale (EPDS) for screening patients in emergency departments, and examine correlates of postpartum depression	2 items querying worry about the food supply and inability to eat the way you should due to lack of money	EPDS 3-item short form	Postpartum depressive symptoms	Maternal age, ethnicity, education, marital status, employment, maternal health problems, health insurance, household income, household size, father’s presence in the home, social support, infant health and health insurance,	Multivariable analyses: Having concerns about food increased odds (aOR 5.5, 95% CI 2.2–13.5) of postpartum depression.
Carter et al. 2011 [43]	General population (15+ years) in New Zealand, included NZ/European, Maori, Pacific, Asian, and other groups. New Zealand Survey of Families, Income, and Employment, 2002–2010	Cross-sectional (18,090)	Examine association between food insecurity and psychological distress	Food security items from measure of individual deprivation (NZiDep): 3 items querying use of food banks and food compromises due to lack of money for food in last 12 months	Kessler-10 scale	Symptoms of psychological distress	Age, ethnicity, legal marital status, family composition, household income, employment, highest level of education, individual-level deprivation	Multivariable analyses: Women who experienced food insecurity were more likely to report moderate to high levels of psychological distress (OR 2.1, 95% CI 1.8–2.4).
Klesges et al. 2001 [68]	Disabled women (65+ years) living in the community in Baltimore, US, primarily White women Women’s Health and Aging Study	Cross-sectional (1001)	Examine prevalence and correlates of financial difficulty acquiring food	Single item, self-perception of food sufficiency “How often does it happen that you (and your husband) do not have enough money to afford the kind of food you should have?” Authors note that such single-item measures have shown validity in discriminating energy intake differences, but have poor sensitivity and underestimate prevalence	Geriatric Depression Scale (GDS),Hopkins Symptom Checklist subscale for anxiety, 20-item perceived quality of life scale	Symptoms of depression, anxiety, quality of life	Age, marital status, and number of household members	Multivariable analyses: In non-white women, depression was associated with financial difficulty accessing food (aOR 1.13, 95% CI 1.04–1.22). This association not significant among white women after adjusting for covariates.
Sharkey et al. 2003 [69]	Women (60+ years) who are homebound (as a result of disability, illness, or isolation), recruited from meal delivery programs in North Carolina, US, included African-American and White women Nutrition and Function Study (NAFS)	Cross-sectional (279)	Examine food sufficiency and association with dietary intake and burden of multiple diseases	Four items adapted from a national nutrition evaluation survey, 2 situations related to lack of food, 2 related to making trade-offs between food and other necessities Authors noted that the items were previously used in a national evaluation of elderly nutrition programs	Geriatric Depression Scale (GDS) 15-item short form	Depressive symptoms	Not applicable	Bivariate analyses: Women experiencing food insufficiency had higher prevalence of 6 or more depressive symptoms (52% vs. 26%, *p* = 0.03) and disease multi-morbidity (74% vs. 41%, *p* < 0.001) compared to those who were food sufficient.
